# Patients with Interstitial Lung Disease Secondary to Autoimmune Diseases: How to Recognize Them?

**DOI:** 10.3390/diagnostics10040208

**Published:** 2020-04-09

**Authors:** Domenico Sambataro, Gianluca Sambataro, Francesca Pignataro, Giovanni Zanframundo, Veronica Codullo, Evelina Fagone, Emanuele Martorana, Francesco Ferro, Martina Orlandi, Nicoletta Del Papa, Lorenzo Cavagna, Lorenzo Malatino, Michele Colaci, Carlo Vancheri

**Affiliations:** 1 Artroreuma S.R.L., Outpatient clinic of Rheumatology associated with the National Health System Corso S. Vito 53, 95030 Catania, Italy; 2 Department of Clinical and Experimental Medicine, Internal Medicine Unit, Cannizzaro Hospital, University of Catania, via Messina 829, 95100 Catania, Italy; malatino@unict.it (L.M.); michele.colaci@unict.it (M.C.); 3 Regional Referral Centre for Rare Lung Diseases, A. O. U. “Policlinico-Vittorio Emanuele” Dept. of Clinical and Experimental Medicine, University of Catania, via S. Sofia 68, pavillon 3 floor 1, 95123 Catania, Italy; eva.fag@virgilio.it (E.F.); emanuele@martorana.email (E.M.); vancheri@unict.it (C.V.); 4 Scleroderma clinic, Department of Rheumatology, ASST G. Pini, 20122 Milan, Italy; francy.pignataro@hotmail.it (F.P.); nicoletta.delpapa@asst-pin-cto.it (N.D.P.); 5 Division of Rheumatology, Hospital IRCCS Policlinico S. Matteo Foundation of Pavia, 27100 Pavia, Italy; gio.zanframundo@gmail.com (G.Z.); veronicacodullo@yahoo.it (V.C.); lorenzo.cavagna@unipv.it (L.C.); 6 Rheumatology Unit, Department of Clinical and Experimental Medicine, University of Pisa, 56126 Pisa, Italy; francescoferrodoc@gmail.com; 7 Department of Experimental and Clinical Medicine, Division of Rheumatology AOUC, University of Florence, 50139 Florence, Italy; martinaorlandi@hotmail.it

**Keywords:** interstitial lung disease, idiopathic pulmonary fibrosis, interstitial pneumonia with autoimmune features, systemic sclerosis, myositis, antisynthetase syndrome, Raynaud’s phenomenon, Sjögren’s syndrome, nailfold videocapillaroscopy, multidisciplinary team

## Abstract

The diagnostic assessment of patients with Interstitial Lung Disease (ILD) can be challenging due to the large number of possible causes. Moreover, the diagnostic approach can be limited by the severity of the disease, which may not allow invasive exams. To overcome this issue, the referral centers for ILD organized Multidisciplinary Teams (MDTs), including physicians and experts in complementary discipline, to discuss the management of doubtful cases of ILD. MDT is currently considered the gold standard for ILD diagnosis, but it is not often simple to organize and, furthermore, rheumatologists are still not always included. In fact, even if rheumatologic conditions represent a common cause of ILD, they are sometimes difficult to recognize, considering the variegated clinical features and their association with all possible radiographic patterns of ILD. The first objective of this review is to describe the clinical, laboratory, and instrumental tests that can drive a diagnosis toward a possible rheumatic disease. The secondary objective is to propose a set of first-line tests to perform in all patients in order to recognize any possible rheumatic conditions underlying ILD.

## 1. Introduction

The diagnostic assessment of patients with Interstitial Lung Disease (ILD) can be challenging, since this condition is associated with several possible diseases [1]. The utility of invasive exams should be also balanced, between the amount of histological information provided and the risk of side effects, mainly in elderly patients or in subjects with a severe disease [2]. Currently, the gold standard in the diagnosis and management of ILD is the Multidisciplinary Team (MDT), in which physicians of several disciplines discuss doubtful cases to reach a confident diagnosis [3]. Lung involvement is common in rheumatic conditions such as vasculitis and Connective Tissue Diseases (CTDs), but the identification of an autoimmune disease underlying ILD can be difficult for the presence of nuanced clinical pictures. Moreover, the lung can be the first manifestation of a CTD (preceding the onset of a definite autoimmune disease by years) as the dominant or even as the sole organ involvement during an autoimmune disease. Obviously, the correct diagnostic assessment of ILD patients has important consequences in prognosis and treatment. Although the rheumatologic evaluation has proved to be effective in reducing invasive exams, currently the rheumatologist figure is not widely present in MDTs [3,4]. The reasons can be numerous: lack of rheumatologists (or of rheumatologists with lung expertise), difficulty in organizing MDTs, and the underestimation of the rheumatologist’s potential role. 

The first objective of this review is to describe the main clinical and serological features, as well as the instrumental evaluation useful for physicians to guide the diagnosis toward a possible autoimmune disease. This review aims to summarize the main clinical and serological features suggestive for an ILD secondary to rheumatic disorders. Rather than describing and collecting all clinical information of rheumatology competence, we tried to emphasize the most important clinical signs, that should also be recognized by non-rheumatology specialists who study and treat patients with ILD associated with Autoimmune Disorders (ADs). 

The secondary aim of this work is to expose our modus operandi for the identification of a possible rheumatic disease in patients with ILD. 

## 2. Clinical Signs

Clinical signs can immediately supply important elements for a diagnosis of autoimmune diseases. However, their actual recognition may require confirmation by rheumatologists with appropriate expertise. 

### 2.1. Arthritis, Arthralgia, and Morning Stiffness

Arthritis is a clinical condition characterized by pain and stiffness of one or more joints due to an inflammatory process. The classical signs of inflammation (particularly the joint swelling) are present with variable degree. Therefore, arthritis is generally a simple diagnosis, even though several conditions may mimic it and should be excluded by a rheumatologist. The patient’s history itself is fundamental during a correct diagnostic work-up. Typically, the joint pain is more intense at rest and during the first hours of the day (h 3–6 a.m.) and may be reduced by movement. Moreover, pain is associated with joint morning stiffness, prolonged more than 30 min up to hours. Conversely, the pain due to osteoarthritis (very common in the general population and not related to ILD) is associated with movement, mainly at the beginning, whereas it is generally absent at rest and overnight. Moreover, in the course of osteoarthritis, joint stiffness lasts few minutes, in any case less than 30 min. Further clinical symptoms could be useful for differential diagnosis, such as pain and stiffness relief with warmth (i.e., shower, paraffin, mud baths) in osteoarthritis. The presence of arthritis per se is not always a sign of chronic inflammatory arthropathy, such as in the case of Rheumatoid Arthritis (RA) that could be linked with ILD. Other pathologic conditions may lead to acute inflammation of one or more joints, such as infectious events, microcrystalline arthritis, or osteoarthritis itself. Hence, clinical presentation and duration are fundamental information to make diagnosis and to differentiate ADs-ILD versus the mere coexistence of two separate pathologic disorders.

Considering the rheumatic conditions that can cause ILD, the most frequent disease characterized by arthritis is RA. Nonetheless, arthritis also has a prevalence of 16% in Sjögren’s Syndrome (SjS) [5], 15–20% in Systemic Sclerosis (SSc) [6], and up to 90% in Antisynthetase Syndrome (ASS) [7]. There is a broad spectrum of presentation ranging from a mono- or oligoarticular form, more common in SSc, to a polyarticular form involving small joints typical of RA. The presence of arthritis justifies a deeper diagnostic search aimed at considering a secondary form of ILD.

Isolated arthralgia is a highly non-specific symptom that may be present in all rheumatic diseases and not necessarily limited to these. The strategy on how to investigate an arthralgia for a possible progression to arthritis is still under discussion. The term “Clinically Suspect Arthralgia” (CSA) has been coined to identify patients with arthralgia for less than a year, which the rheumatologist suspects may progress toward arthritis on the basis of his personal clinical experience [8]. CSA represents about 7% of patients with arthralgia who refer to an outpatient clinic of rheumatology and, of these, 20% develop RA during the follow-up [9]. The characteristics of arthralgia at risk of RA are the following: joint symptoms onset < 1 year, symptoms located in metacarpophalangeal joints, morning stiffness ≥ 60 min, most severe symptoms present in the early morning, presence of a first-degree relative with RA, difficulty in making a fist, positive squeeze test of metacarpophalangeal joints [10]. The presence of at least 4 parameters to define CSA shows 70.5% sensitivity and 93.6% specificity.

Arthralgia affecting the scapular and pelvic girdles in association with a severe morning stiffness and elevated C-Reactive Protein (CRP) and/or Erythrocyte Sedimentation Rate (ESR) could orientate toward a diagnosis of Polymyalgia Rheumatica (PMR). The current PMR criteria show a high sensitivity, but a low specificity in the distinction between PMR and other inflammatory, mainly autoimmune, diseases [11]. Sometimes, the distinction between PMR and late-onset RA or oligo-amyopathic myopathy can be very difficult. Recently, cases of association between ILD and PMR have been reported, although the possible association between the two pathologies needs further investigations [12]. Respiratory symptoms in the course of PMR should be investigated in relation to possible ILD, especially if asthenia, arthralgias, and morning stiffness do not show response to low-dose steroid therapy. In these cases, the diagnosis should be re-evaluated and the possible presence of CTD should be taken into consideration.

### 2.2. Sicca Syndrome and Glandular Swelling

Xerophthalmia and xerostomia are very common symptoms. The prevalence in the general population is 5–30% and 0.02–40%, respectively, with an increase correlated with the age of the population [13,14].

Patients with CTDs frequently report these symptoms that represent the main features of SjS. CTDs are only one of the possible causes of dryness and are among the rarest, considering that the prevalence of SjS is 0.03% of the general population [15]. Sicca syndrome is also described in 25% of patients with RA [16] and in up to 71% of those with SSc [17], not necessarily associated with SjS. 

About one-third of patients with SjS experience episodes of swelling of the salivary or lachrymal glands [18]. It has been demonstrated that parotid swelling can anticipate the onset of sicca syndrome and subsequent confirmation of the diagnosis of SjS by up to 14 years [19]. The evaluation of a glandular swelling is relevant, considering that 49% of patients with SjS-ILD did not show sicca symptoms at the onset of the disease [20]. Glandular swelling is considered a criterion of disease activity in SjS and should be clinically evaluated. Nonetheless, ultrasounds provide useful information on the glandular structure, helping in the disease diagnosis and stadiation [21].

In the course of SjS, parotid and submandibular salivary glands are the most common sites of lymphoma of the mucosa-associated lymphoid tissue, which typically occurs as a persistent and hard monolateral swelling and has an important impact on prognosis [22].

### 2.3. Raynaud’s Phenomenon, Digital Ulcers, and Pitting Scars

**Figure 1:**
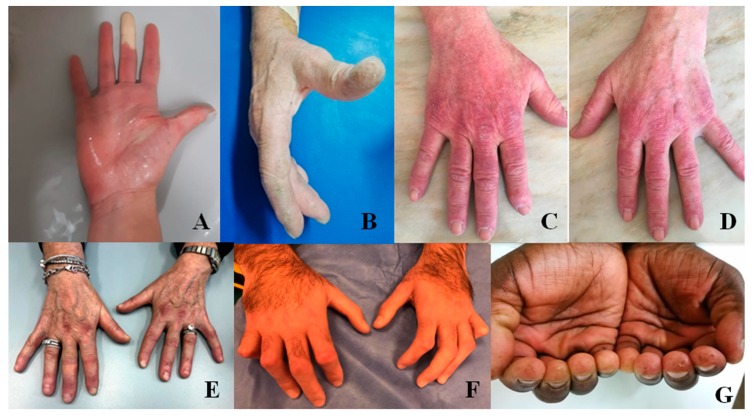
The hands in patients with Interstitial Lung Disease (ILD) secondary to rheumatic conditions. (**A**): Raynaud’s phenomenon in ischemic phase during a provocation test; (**B**): mechanic’s hands; (**C**,**D**): Gottron’s signs; (**E**): Gottron papules; (**F**): sclerodactyly with clawed shape in systemic sclerosis; (**G**): pitting scars in a patient with systemic sclerosis.

Raynaud’s phenomenon (RP) owes its name to Maurice Raynaud, who described it for the first time in his doctoral thesis in 1862. RP is characterized by a change of the color of the fingers in 3 subsequent phases: white (during ischemic phases), blue (during hypoxic phases), and red (during revascularization) (Figure 1). At least the first two phases are needed to recognize the presence of RP [23]. 

This condition affects 3–5% of the general population with a marked increase in prevalence in cold regions [24]. RP is usually generated by an external stimulus, mainly the exposure to cold, but also emotional factors and vibrations. It may be aggravated by cigarette smoking, caffeine intake, or drugs including non-selective beta-blockers and chemotherapy, among others. In response to cold, subjects with RP have a marked sympathetic-mediated vasoconstriction involving the pre-capillary sphincters, leading to the white coloring of the skin. In normal conditions, the reduction of the capillary flow determines the activation of the endothelium with dilatation of the upstream vessels which guarantees the nutritional flow downstream [25]. In RP, this mechanism of protection is less efficient. Thus, RP was subclassified into primary, where the vasospasm is not sufficient to generate a downstream damage, and secondary, where, in association with structural damage and endothelial dysfunction, damage can occur.

Up to 90% of RP cases are classified as primary [26]. Secondary RP is observed in more than 90% of cases of SSc or Mixed Connective Tissue Disease (MCTD) and in up to 2/3 of patients with ASS, and it is considered in the classification criteria of these conditions [27,28]. RP is also observed in 18–45% of patients with Systemic Lupus Erythematosus (SLE), 20% with Dermatomyositis (DM)/Polymyositis (PM), and 10–20% with RA [24]. Nailfold videocapillaroscopy (NVC) and autoantibody panel play a pivotal role in the recognition of secondary RP. 

The transition from primary to secondary RP occurs in 3.2% of patients per year with a mean time of 10 years from the onset of RP [29]. This supports the importance of a tight follow-up. 

The presence of cyanosis at the extremities without the ischemic phase of RP is defined as “acrocyanosis”, a clinical entity frequent in the general population that could also be considered part of the clinical picture of a CTD. 

Digital Ulcers (DUs) represent the clinical manifestation of microvascular damage. It is estimated that approximately 50% of patients with SSc develop at least one DU during their clinical history. Moreover, the presence of DUs is a classification criterion of SSc and is associated with more severe subsets of the disease [27,30].

DUs can be extremely painful and have an important impact on patients’ quality of life [31]. They are frequently complicated by infection and the presence of osteomyelitis is observed in 42% of patients with infected DUs [32]. Healing is very slow with a mean time of 76 days in pure DUs, 93 in calcinosis, and 281 in gangrene [33]. 

Digital Pitting Scars (DPSs) are defined as “pinhole-sized digital concave depressions with hyperkeratosis” [34]. Observed in 34–53% of cases [30], they have greater weight than DUs in the classification criteria for SSc (3 vs. 2 points) [27]. Unlike DUs, their localization is not limited to the fingertips, but they are also present laterally, including on the radial surface of the second and third finger and the ulnar surface of the first finger. The factors that seem to be determinant in the genesis of DPSs are ischemia, cold exposure, and micro-traumatisms [34]. Usually not painful, they tend to heal faster than DUs (25 days) [33].

It is important to evaluate the possible coexistence of other signs, considering that DUs and DPSs commonly occur in the presence of RP or skin sclerosis and, therefore, in these cases, they strongly support the diagnosis of SSc. 

### 2.4. Puffy Hands and Skin Sclerosis

Puffy Hands (PHs) are often considered the first sign of SSc after the onset of RP [35], but they are also observed in Undifferentiated CTD and MCTD and considered an additional criterion in the definition of Very Early Diagnosis of SSc (VEDOSS) [36]. Moreover, their presence satisfies 2 of the 9 points necessary for the classification of SSc [27]. This phase is characterized by the presence of edema of the hands that may remain stable or evolve, even after a long time, toward fibrosis. A state of persistent hypoxia, the presence of proinflammatory and profibrotic cytokines, fibroblast activation, oxidative stress, microvascular damage, and ineffectiveness of neoangiogenic mechanisms gradually cause the appearance of fibrosis [37]. Skin fibrosis is the hallmark of SSc. In particular, the presence of skin fibrosis of both hands extending proximal to the metacarpophalangeal joints is a sufficient classification criterion [27]. Progressively, fibrosis can become so severe as to cause contracture of the joints with a serious impact on the patients’ quality of life. This has a prevalence of 31% of patients with SSc [38]. 

The presence of skin sclerosis on the extremities (generally hands) and face identifies the limited cutaneous subset of Ssc, while the involvement of the trunk and the proximal limbs defines the diffuse one [39].

The degree of cutaneous involvement can be measured with the modified Rodnan skin score: the obtained value correlates with disease activity, disease severity, and mortality in the course of SSc [40]. 

### 2.5. Gottron’s Papules and Gottron’s Sign

Described for the first time by Gottron in 1930, this cutaneous manifestation consists of violaceous non-palpable macules (Gottron’s sign) or raised papules (Gottron’s papules) on the surface of bone prominence (Figure 1) [41]. Characteristically, the lesions occur over the metacarpophalangeal and interphalangeal joints and more rarely in the elbows, knees, and/or feet [42]. Gottron’s papules have been observed in about 50% of patients with ASS [43] and in up to 87% of patients with DM and are considered a pathognomonic sign of this disease [44,45].

In view of the pathognomonicity of Gottron’s sign, it is interesting to note that the sign is included among the classification criteria for Interstitial Pneumonia with Autoimmune Features (IPAF) [46]. On the contrary, in the prospective cohort of IPAF no case showed Gottron’s sign [47]. It is possible to imagine that the simultaneous presence of Gottron’s sign and ILD would lead the MDT toward the definition of CTD-ILD. 

### 2.6. Mechanic’s Hands and Hiker’s Feet

Mechanic’s Hands (MHs) was described for the first time in 1979 as a hyperkeratotic and non-pruritic eruption of the hands with scaling, fissuring, and hyperpigmentation. It typically shows a symmetrical involvement of the ulnar surface of the first finger and the radial surface of the others, principally the second and third finger [48]. These alterations resemble a manual laborer’s hands, but only 1 out of the 8 patients observed actually did manual work. Four of these patients had a diagnosis of MCTD, 3 of DM, and 1 of SLE, but all manifested a myositic involvement. The presence of MHs is observed in 40% of patients with DM and in 30–70% of those with ASS [49] and is part of both classification criteria for ASS proposed in 2010 and 2011 [28]; however, the clinical importance of MHs is still under study. Indeed, MHs appear to be associated with an increased risk of systemic involvement, especially lung, and a lower risk of malignancy in DM [50]. 

Skin lesions similar to those seen on the hands can also be observed in the feet. In this case, the hyperkeratosis is associated with cracking and dryness and involves the plantar surface, including the toes, reminiscent of the feet of long-distance walkers. The term “hiker’s feet” was chosen to identify this cutaneous sign, observed in DM and ASS and associated in 90% of cases with MH [51]. 

### 2.7. Heliotrope Rash

Its name is due to the typical color that recalls the flower *Heliotropium*. It is a violaceous erythema involving the upper eyelid and periorbital tissue, that can also extend to the cheeks and nasolabial fold. Frequently, the rash is associated with overt edema up to reducing eyelid opening. This is considered a characteristic sign of DM, and it has also been observed in 14% of patients with ASS [45,52].

### 2.8. Shawl Sign, V-Sign, and Holster Sign

They consist of a red rash, which may be either flat or raised, involving the upper back, shoulders and arms (shawl sign), the skin in the anterior area of the neck and upper chest with a “V”-shaped pattern (V-sign), or the external area of the hip (holster sign) [53]. They are considered characteristic signs of DM [45]. 

### 2.9. Telangiectasias

These are enlarged capillaries visible on the skin surface. They occur characteristically in SSc, mainly in limited cutaneous form, so that they are included in the classification criteria [27]. In SSc, telangiectasias are commonly localized in the face, lips, hands, and inside the mouth and might be associated with the presence of pulmonary arterial hypertension [54]. Telangiectasias can also be observed in DM and ASS, commonly in periungual areas, but also in periorbital and gingival areas [53,55]. Telangiectasias are considered in the clinical domain of the classification criteria for IPAF, even if limited to those on the palmar surface [46]. For the purpose of the definition of IPAF, it would seem useful to also extend the location of telangiectasias to the other areas commonly involved during CTDs.

### 2.10. Calcinosis

Calcinosis is defined as the accumulation of insoluble calcium salts in various tissues. Classically, calcinosis is classified into five classes: (i) metastatic calcinosis, often associated with malignancies, with abnormal serum level of phosphorous and calcium that commonly affects the wall of arteries and internal organs; (ii) tumoral calcinosis, initially limited to a rare form of genetic disease and characterized by a high level of serum phosphorous and normal level of calcium, then generally understood as any form of large calcification; (iii) dystrophic calcinosis, commonly observed in ADs, particularly in SSc, DM, and MCTD. In this case, there is a normal serum level of calcium and phosphorous, and the areas principally involved are skin and subcutaneous tissue; (iv) idiopathic calcification, that occurs in healthy individuals with normal serum levels of calcium and phosphorous; (v) calciphylaxis, typical of patients with chronic renal failure, characterized by an alteration of the serum levels of calcium and phosphorous and involvement of the vessels that can lead to ischemia [56]. 

Calcinosis affects approximately 25% of patients with SSc, 20% of adults with DM, and up to 70% of those having the juvenile form [57,58]. The pathogenesis of calcinosis is poorly understood. Chronic inflammation, local microtraumas, and hypoxia are thought to play an important role [59]. The most common sites are the skin and subcutaneous tissue in SSc and proximal limb muscles in DM [60]. However, calcinosis can occur in other areas, including paravertebral sites, and reach the size of tumoral calcinosis [61]. Calcifications occur in the chronic phase of disease—in SSc, about 9 years after onset and more commonly in limited cutaneous SSc [62]. Anecdotally, the calcification anticipates the diagnosis of SSc [61]. 

Another type of subcutaneous formation that can be found at the bone prominence is the rheumatoid nodule. Even though not frequently reported in clinical practice, it is characteristic of long-standing RA; thus, it could represent a useful sign to address the clinician toward the rheumatological diagnosis.

### 2.11. Muscle Weakness

Muscle weakness is a common sign of myopathies. Ten different patterns have been described that should guide the examiner in the diagnosis. In the course of inflammatory myopathies, muscle weakness is often associated with myalgia and principally manifests three patterns: (i) proximal “limb-girdle” weakness that symmetrically affects the proximal muscles of arms and legs, with less involvement of the distal muscle. This pattern is the most frequent and common to many myopathies and is therefore not specific; (ii) prominent neck extensor weakness, also called “dropped head syndrome”, where amyotrophic lateral sclerosis and myasthenia gravis should also be considered in the differential diagnosis; (iii) episodic pain, weakness, and myoglobinuria, mainly linked to intense physical exercise in an untrained subject, but it has rarely been described as not related to physical exercise in subjects with DM/PM [63]. Muscle weakness can also be observed during PMR and, therefore, requires subsequent examinations in order to be defined. 

### 2.12. Dysphagia

During the course of PM, patients may be affected by dysphagia, due to the muscle involvement of pharynx and the upper third of the esophagus. This condition may become severe because of the impairment of patients’ ability to eat and drink. 

Another kind of dysphagia regards the smooth muscular wall of the lower part of esophagus in SSc patients. The visceral hypotonia and dyskinesia develop slowly during the patients’ clinical history, affecting the ability to swallow and the quality of life in various degrees. 

Finally, SjS patients may also complain of dysphagia because of the dryness of the mouth and pharynx exacerbated during the meal.

### 2.13. Fever of Unknown Origin 

An unexplained fever may be present in the course of all ADs related to ILD. The sign is highly non-specific and needs to be properly investigated. Generally, fever secondary to CTDs is < 38 °C, while higher temperatures should be always investigated to exclude concomitant infectious diseases.

## 3. Laboratory Exams

Laboratory exams can play a pivotal role in the diagnostic assessment of ILD, supporting a specific diagnosis of ADs, above all in patients with nuanced accompanying clinical features. For convenience, we distinguish general exams from autoimmune exams.

## 4. General Laboratory Exams

A panel of general laboratory tests usually gives useful information regarding the level of inflammation, eventual hepatic or renal dysfunction, CTD-related alterations, and possible comorbidities. This panel should consider a complete blood test, ESR, CRP, complement fractions C3 and C4, Serum Protein Electrophoresis (SPEP), urine test, creatinine, Alanine Aminotransferase (ALT), Aspartate Aminotransferase (AST), Creatinine Phosphokinase (CPK), Lactic Dehydrogenase (LDH), myoglobin, and aldolase. 

A complete blood count should be always carried out in all ILD patients. It can be useful to assess the level of hemoglobin in order to regulate the use of drugs with a high risk of myelo-suppression. Anemia may be secondary to chronic inflammation. In this view, the increased number of White Blood Cells (WBCs) and their formula can suggest inflammation (sustained by ADs or infections). The reduction of platelets or WBCs may be useful for diagnostic purposes, given that this is considered in the diagnostic criteria for SLE [64].

Increased levels of ESR, CRP, C3, and C4 are associated with inflammation, but they are not able to distinguish between infectious or autoimmune origin. The dosage of procalcitonin may be helpful in the former case. Moreover, they can be burdened by several cases of false positives or negatives and their value should be carefully evaluated in the absence of a concordant clinic [65]. However, ESR and CRP are commonly high in autoimmune inflammation, especially in RA, in which they are included as criteria [66]. The reduction of the fractions of complement C3 and C4 can be related to active SLE (therefore included in the classification criteria) and with Anti-Neutrophil Cytoplasm Antibody (ANCA)-Associated Vasculitis (AAV) [67,68]. SPEP is able to provide interesting information. The finding of an increased portion of α2 globulins is considered a sign of inflammation, and the study of γ globulins in SPEP is also of some interest in ILD patients. Although hypergammaglobulinemia is not included in any classification criteria of CTDs, it has commonly been found in these patients, mainly in SLE and SjS [69]. On the contrary, hypogammaglobulinemia is a marker of common variable immunodeficiency, a condition that can produce ILD in the lung, mainly resembling an advanced stage of sarcoidosis in High-Resolution Computed Tomography (HRCT) [70]. 

Urine test, creatinine, and transaminases are useful in the assessment of the kidney and liver functions in order to manage possible treatments, such as Disease-Modifying Anti-Rheumatic Drugs (DMARDs) or antibiotics. The urine test diagnostic value is essential for the recognition of proteinuria, hematuria, and/or chronic renal failure, potentially linked to SLE or AAV.

AST, LDH, CPK, myoglobin, and aldolase are muscular enzymes, generally increased in patients with an active inflammatory muscular involvement in Idiopathic Inflammatory Myopathies (IIMs). PM, DM, and ASS are included in this group, being conditions associated with potentially severe ILD [71,72]. During IIMs, one or more of these enzymes can have high serum levels, but they can also be elevated in other conditions such as hepatic injury or ILD itself [73]. Therefore, it could be reasonable to consider second-line tests to support a suspected diagnosis of IIMs [74]. 

## 5. Autoantibodies

The search of autoantibodies is very useful for the diagnostic assessment of ILD patients, due to the general high association with CTDs. They can reflect the autoimmune activity of B cells, but their positivity should be always considered in association with the clinical picture. Indeed, a single autoantibody positivity without appropriate clinical findings may be not related to an established disease or may be stochastically positive or anticipate the disease onset. In our opinion, we can distinguish first-line and second-line autoimmune exams.

## 6. First-Line Autoimmunity Exams

In this class, we consider Antinuclear Antibodies (ANAs), Rheumatoid Factor (RF), Anti-Citrullinated Protein Antibody (ACPA), Double-Stranded DNA (DsDNA), ANCA, and the Extractable Nuclear Antigen (ENA) profile. The latter group, generally available in commercial kits, includes anti-Ro/SSA, anti-La/SSB, anti-Ribonucleoprotein (RNP), anti-Sm, anti-topoisomerase I (Scl70), and anti-Jo1.

ANAs can be positive in all ADs, typically in SLE [64], and they are also considered in the IPAF criteria, a research classification similar to the concept of undifferentiated CTD. The inclusion of ANAs in these criteria is reasonable, considering that they may precede the disease onset by 5 years [75]. However, ANA positivity can be found in a large number of conditions not related to ILD and even in normal subjects [76,77]. Therefore, as well as for all potentially autoimmune items, they should be considered along with the other clinical features for every patient. In addition to the titer, the pattern of ANA positivity could also be very useful [78]. Indeed, a centromeric pattern is pathognomonic for the presence of Anticentromere Antibodies (ACAs). Of great interest is the nucleolar and cytoplasmic positivity, because these can suggest positivity for Antisynthetase Antibodies (ATSAs) regardless of the seric titer and should be studied in depth, looking for the presence of Myositis-Associated or Myositis-Specific Antibodies (MAAs and MSAs).

RF and ACPA are specific antibodies for the diagnosis of RA [3]. Both these antibodies can be positive several years before the disease onset and the combined positivity seems to have 100% risk of developing RA within 5 years [79]. However, their diagnostic value is different. RF can be positive also in other conditions, not necessarily of autoimmune origin, and even in up to 25% of the general population [80]. Therefore, RF positivity is generally considered as low and high titers using two times the upper limit as the cutoff for IPAF criteria, and three times the limit for RA ones [46,66]. ACPAs are more specific for RA than for RF, but they can be positive in about 1% of normal subjects also (who are at risk for RA in any case) [79,80,81,82]. ACPAs are also interesting for the pathogenesis of ILD: the lung could be the first site of injury in RA [83]. Increased evidences support citrullination of lung peptides due to cigarette smoking and other environmental triggers, as well as possible microbial molecular mimicry. These conditions could lead to the production of ACPAs and the subsequent development of RA. 

DsDNAs are highly specific for SLE and their presence in healthy subjects is very uncommon [64,84]. Despite the utility in the diagnosis and prognosis of SLE, the utility in the management of ILD patients seems to be limited, considering the rarity of ILD-SLE [72]. Moreover, DsDNAs were considered in the serological criteria of IPAF. In prospective studies of IPAF patients, these autoantibodies were rarely recognized, even at low titer, and not confirmed during follow-up, thus suggesting a possible false positivity [85,86]. 

ANCAs are a group of autoantibodies specific for AAV, but they can be positive in several other ADs [87]. They are generally divided into proteinase-3 (PR3), specific for Granulomatosis with Polyangiitis (GPA); Myeloperoxidase (MPO), more specific for Microscopic Polyangiitis (MPA) and Eosinophilic Granulomatosis with Polyangiitis (EGPA); and atypical ANCA [87]. Despite the abovementioned trend of specificity for AAV, ANCAs can be positive in several other conditions (infections, CTD, inflammatory bowel disease, some of which are able to justify ILD). Moreover, EGPA with PR3-ANCA positivity, as well as GPA associated with MPO-ANCA are not uncommon [88]. For this reason, ANCAs are not currently included in any classification criteria, neither for AAV nor for IPAF [89]. However, in recent studies, patients with Idiopathic Pulmonary Fibrosis (IPF) and ANCA positivity developed vasculitis (MPA and GPA) [86,90]. This is in line with the current knowledge that recognizes the Usual Interstitial Pneumonia (UIP) pattern as the most common ILD in AAV [91]. For this reason, we considered it appropriate to include ANCA in the first-line exams for the diagnosis of ILD patients, mainly if with a UIP pattern or imaging resembling sarcoidosis [92]. 

As already mentioned, the ENA profile includes autoantibodies that are highly specific for several CTDs. However, their utility in ILD patients has different values. Anticentromere Antibody (ACA, also named CENtromere Protein B, CENP-B) and anti-Scl70 are associated with SSc [93], but the former is protective for ILD, while the latter is associated with severe forms [94]. Anti-Sm and Anti-RNP can be present in several CTDs, but are specific for SLE, while anti-U1-RNP is specific for MCTD [95]. Anti-RNP is generally associated with mild myositis and ILD [96]. Anti-SSA and anti-SSB are associated with SjS, but the latter antibody was excluded by new classification criteria for SjS [97]. In fact, anti-SSB alone without accompanying anti-SSA is uncommon and unrelated to SjS [98]. Anti-SSA can be directed to the subunit of 52 or 60 kD [99]. Both are specific for SjS, but they can be found in several other CTDs, especially SSA52 kD, which is frequently associated with IIMs [96]. It should be taken into consideration that anti-SSA can be positive at ANA negative for the loss of anti-SSA60 kD during preparation, but also because SSA52 kD recognizes a cytoplasmic antigen [96]. SSA52 kD is mainly associated with ILD in both IIMs and SjS [69]. The ENA profile also includes anti-Jo1, the most frequent antisynthetase antibody and the unique one included in ENA commercial kits. It recognizes histidyl-tRNA synthetase, a cytoplasmic protein; therefore, similarly to anti SSA52 kD, it can be positive with ANA negative [96]. Anti-Jo1 is associated with arthritis, myositis, and ILD in IIMs and mainly ASS [100,101].

## 7. Second-Line Autoimmunity Exams

A number of other, generally rare, MSAs and MAAs can be associated with ILD, the largest part belonging to the family of IIMs [96]. 

Among the MAAs, Pm/scl75 kD and Pm/scl100 kD (based on the protein recognized) are found in IIMs, overlap syndromes, and SSc, generally seronegative for Scl70 or ACA [102] and are associated with mild myositis and ILD with better outcome compared to Scl70 + SSc [103]. These autoantibodies were not found in healthy subjects [104]. Another uncommon antibody that can be found in several CTDs is anti-Ku. Patients with this antibody can have myositis and ILD, the latter refractory to steroids [105]. Moreover, Anti-Mitochondrial Antibody (AMA) M2 antibody, greatly specific for primary biliary cholangitis, can be associated with IIMs and ILD [106], but no evidence is reported in literature regarding the clinical features of AMA M2 + ILD patients. 

Among MSAs, rare ATSAs different from anti-Jo1 are included (e.g., PL7, PL12, EJ, OJ, Ks). Regarding cytoplasmic antigens, they can be positive regardless of ANA. They are generally associated with amyopathic or mild form or myositis, but potentially severe ILD. Increased evidences suggest a possible specific disease subset for each antibody specificity [107]. Anti-Mi2 and antibodies to small ubiquitin-like modifier activating enzyme (anti-SAE) are associated with the diagnosis of juvenile DM and skin involvement in DM, respectively, but both myositis and ILD seem to have a good prognosis when associated to these antibodies [108]. The most dangerous MSAs can be considered anti-Melanoma Differentiation-Associated 5 gene (anti-MDA5), generally associated with clinically amyopathic DM and rapidly progressive ILD [109]. Several other MSAs can be useful for the diagnosis of IIMs, but their role in the assessment of ILD is not currently studied in depth [96]. Some of the most common are TIF1, anti-signal recognition particles, anti-SAE, antibody to 3-hydroxy-3methyl-glutaryl-Coenzyme A Reductase (anti-HMGCR). 

A distinct mention should be made for Antiphospholipid Antibodies (APLAs), anti-cardiolipin antibodies, and anti-β2glicoprotein I antibodies. They are specific for Antiphospholipid Syndrome (APS), alone or associated with other CTDs (mainly SLE), but they are also present in 1%–5% of healthy subjects [110]. No associations are currently reported regarding ILD and APL. However, it should be considered that Idiopathic Pulmonary Fibrosis (IPF) shows a pro-thrombotic status and vascular events are common [111]. In this disease, Lupus Anticoagulant (LAC) is reported positive in about 21% of patients [112], a proportion significantly higher compared to what is reported in the general population. LAC is reported also in 35% of SLE patients and is a diagnostic criterion for this condition [64,113], however, it is hard to suppose an overlap with SLE in these patients given the lack of other features. A possible overlapping condition between IPF and APS is an argument of interest.

**Figure 2:**
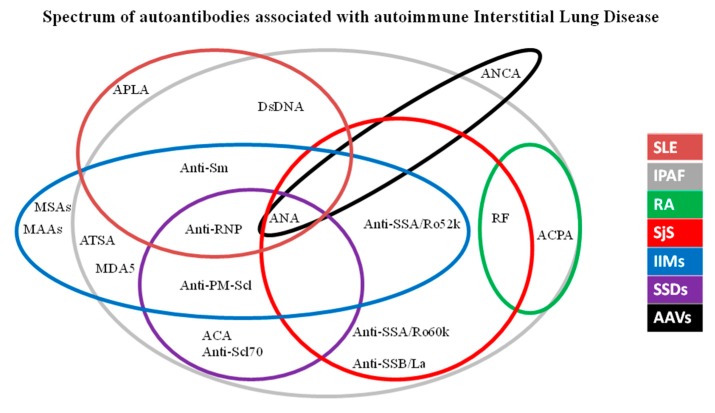
Spectrum of autoantibodies associated with autoimmune interstitial lung disease. Legend: AAVs: ANCA (Anti-Neutrophil Cytoplasm Antibody)-Associated Vasculitis; ACPA: Anti-Citrullinated Protein Antibody; ANA: Antinuclear Antibodies; APLA: Anti-phospholipid antibodies; ATSA: Anti-T-RNA-synthetase antibodies; IIMs: Idiopathic Inflammatory Myopathies; IPAF: Interstitial Pneumonia with Autoimmune Features; MAAs: Myositis-associated antibodies; MSAs: Myositis-specific antibodies; RA: Rheumatoid Arthritis; RF: Rheumatoid Factor SjS: Sjögren’s Syndrome; SSDs: Scleroderma Spectrum Disorders.

Figure 2 shows the main autoantibodies useful for the diagnosis of autoimmune ILD.

## 8. Instrumental Evaluation

Several instrumental exams are able to support a diagnosis of AD underlying an ILD, however, they are not always available in an outpatient setting. In this section, we propose a set of first- and second-line instrumental exams that are useful in the assessment of ILD.

## 9. First-Line Instrumental Exams

NVC is a useful tool to study in vivo the density and morphology of capillaries of the fingers through a magnification of 200 folds. NVC represents an easy, non-invasive, and non-expensive technique, generally used to evaluate patients with RP in order to detect a possible SSc [114]. The most important parameters for the diagnosis are the presence of giant capillaries (capillaries with a diameter ≥50 μ) and Avascular Areas (AAs, distance between two capillary loops ≥ 500 μ) [114]. NVC in SSc has proved to be useful not only in diagnosis but also in prognosis. The Number of microhEMOrrages (NEMO score) is useful to assess the disease activity in SSc, whereas the mean number of capillaries and AAs can be helpful to stratify the risk of developing complications such as DUs [115,116,117,118]. Recently, a pathologic NVC with scleroderma pattern was also found in patients with IIMs without RP [119], and the presence of bushy capillaries was associated with the diagnosis of IIMs [120]. Therefore, NVC can be a useful tool to assess ILD patients in order to select patients in whom it can be appropriate to look for MAAs and MSAs.

The diagnosis of SjS, generally suggested by the presence of sicca syndrome, can be studied by salivary gland ultrasound or sialo-scintigraphy; according to the current criteria, impairment in the glandular function should be demonstrated [97]. In fact, the ocular staining score should be assessed by experienced ophthalmologists, whereas Schirmer’s Test (ST) and Unstimulated Salivary Flow Rate Test (USFRT) can be performed easily in an outpatient clinic assessing ILD [121]. ST is a simple test aimed to measure lacrimal production in 5 min through a measured strip in the lower eyelid. A production of <5 mm in 5 min is considered pathological [122]. USFRT can assess the production of saliva by inviting patients to collect their saliva by passive drool in a measured tube in 5 min. The test is considered positive for a production < 0.1 mL/min [123]. 

Pulmonary Function Tests (PFTs) have a role in the follow-up rather than in the diagnosis of ADs-ILD. Forced Vital Capacity (FVC) and Diffusion Lung Capacity for Carbon Monoxide (DLCO) can describe the severity of restrictive lung damage. This is true mainly for RA, while in scleroderma spectrum disorders and IIMs, conflicting results are reported in the literature regarding the possibility that PFT could appropriately assess the evolution of lung damage and the response to treatment [72]. DLCO is in fact undermined by the frequent presence in these conditions of pulmonary artery hypertension associated with ILD. 

**Figure 3:**
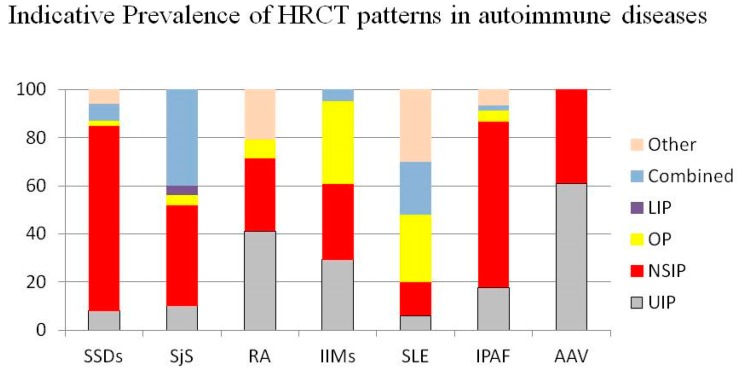
Legend: AAV: ANCA-Associated Vasculitis; LIP: Lymphocytic Interstitial Pneumonia; IPAF: Interstitial Pneumonia with Autoimmune Features; IIMs: Idiopathic Inflammatory Myopathies; NSIP: Non-Specific Interstitial Pneumonia; RA: Rheumatoid Arthritis; SLE: Systemic Lupus Erythematosus; SjS: Sjögren’s Syndrome; SSD: Scleroderma Spectrum Disorders; UIP: Usual Interstitial Pneumonia.

High-Resolution Computed Tomography (HRCT) should be considered a first-line exam for the assessment of ILD patients. Non-Specific Interstitial Pneumonia (NSIP) is the most frequent ILD pattern in CTDs with lung involvement, but all patterns can be observed. UIP is the most common pattern in ILDs associated with RA and AAV, however it is also present in about 10% of ILD-SjS frequently before the onset of sicca syndrome and in the late stage of SSc. Lymphocytic interstitial pneumonia is a rare pattern but is closely related with the diagnosis of SjS. Combined patterns are also common, especially for NSIP-OP. The latter pattern is a common feature of IIM-ILD [124,125,126,127,128,129,130,131]. Figure 3 shows an indicative proportion of the frequency of each HRCT pattern for each rheumatic disease [47,124,125,126,127,128,129,130,131]. The proportions reported can widely vary in the studies, depending from the criteria used for the patients’ enrollment and/or HRCT classification, as well as the stage of the disease at the time of the study. HRCT may also be useful as a prognostic factor in the assessment of ILD severity and progression. Emerging evidences support the role of many quantification scores in the evaluation of prognosis and response to treatment in several CTDs [132,133,134]. 

## 10. Second-Line Instrumental Exams

Several other instrumental exams can be useful in the diagnostic assessment of ILD patients with suspected AD. The most important is biopsy, performed depending on the tissue involved. Lung biopsy can be useful to assess the ILD pattern with more precision, but the acquired information should be balanced with the risk of the procedure [2]. It should be taken into account that, although small studies have reported histological differences between the UIP pattern in RA and IPF (fewer fibroblastic foci and more CD4+ cells in RA), no evidences strongly support a confident differential diagnosis between these two conditions [135]. A similar consideration should be made for sarcoidosis. Indeed, up to 35% of these patients show non-caseating necrotizing granulomas; so, for them a possible alternative diagnosis of AAV or RA should be considered [136]. Minor salivary gland biopsy can be very useful in the diagnosis of ILD underlying SjS, mainly considering the possible seronegative subset of these patients [69]. The kidney and the upper and lower respiratory tract may be involved by inflammation during AAV, but about 30% of histological exams of the respiratory tract can give a false negative result [137]. Muscle biopsy may be useful for the diagnosis of IIMs and the differential diagnosis among the forms including them [138]. However, several subsets of ILD-IIMs had small muscular involvement and, therefore, it could be useful to perform several exams (e.g., echography, electromyography, magnetic resonance imaging) in order to choose the appropriate tissue to collect for a confident diagnosis. 

Finally, kidney biopsy can be considered for the diagnostic definition of ILD patients with suspected SLE and/or AAVs.

## 11. Conclusions

The differential diagnosis of conditions underlying ILD is a fascinating topic involving many physicians of different specialties. Currently, the gold standard for the diagnostic assessment of ILD is MDT and the presence of rheumatologists among them has proved to be useful to reduce the need for invasive exams [4,139]. The role of rheumatologists in MDT is probably more important considering the possible selection bias in the study of ADs-ILD. In fact, AD-ILD patients can refer to the specialist who is perceived as more useful for the clinical condition. In this hypothesis, patients with mild ILD can refer to a rheumatologist, while ILD patients with poor rheumatologic symptoms could be directed to pulmonologists. Several conditions (e.g., SjS, AAVs, RA) can have ILD as the main clinical manifestation of the disease. Moreover, ILD can be the first manifestation of the disease, raising questions about a possible pathogenic role of the lung as an autoimmune source. This is one of the reasons that led to the definition of the IPAF criteria, in order to select patients with primary ILD at risk of developing ADs or those who harbor a subclinical CTD. 

**Figure 4:**
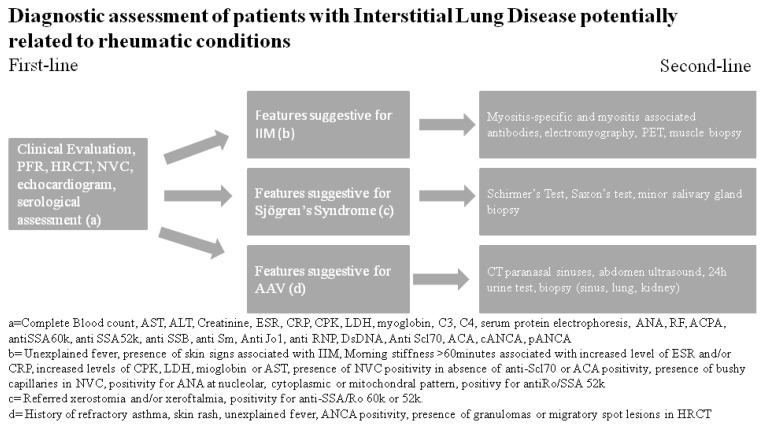
Our methodology in the diagnostic assessment of patients with interstitial lung disease. Legend: AAV: ANCA-Associated Vasculitis; ALT: Alanine Transaminase; ACA: Anticentromere Antibody; ACPA: Anti-Citrullinated Protein Antibody; ANA: Antinuclear Antibody; ANCA: Anti-Neutrophil Cytoplasmic Antibody; AST: Aspartate Transaminase; C3 and C4: Complement Fraction 3 and 4; CPK: Creatine Phosphokinase; CRP: C-Reactive Protein; DsDNA: Double-Stranded DNA; ESR: Erythrocyte Sedimentation Rate; HRCT: High-Resolution Computed Tomography; LDH: Lactic Dehydrogenase; NVC: Nailfold Video Capillaroscopy; PET: Positron Emission Tomography; PFTs: Pulmonary Function Tests; RF: Rheumatoid Factor.

Close collaboration between rheumatologists and pulmonologists, both experienced in the management of ILD, is therefore useful for the assessment of these patients, but currently it is not widely diffused and standardized. In this review, we report our approach in Figure 4. The first step is a careful clinical examination performed by both pulmonologists and rheumatologists. All patients should undergo to HRCT, NVC, PFTs, and a first-line serological assessment. Based on the results obtained, patients can perform second-line exams as reported in the figure. After the study, patients with inconclusive diagnosis could be discussed within the MDT. 

Finally, it is auspicious that the growing knowledge in the field and the interest to better define complex clinical pictures led different specialists to gather together their know-how into shared recommendations in order to manage autoimmune ILD patients as well as possible.

## Abbreviations

Alanine Aminotransferase	ALT
ANCA-Associated Vasculitis	AAV
Anti-3-hydroxy-3methyl-glutaryl-Coenzyme A Reductase	Anti-HMGCR
Anti-Citrullinated Protein Antibody	ACPA
Anti-Neutrophil Cytoplasm Antibody	ANCA
Anti-Small ubiquitin-like modifier activating enzyme	Anti SAE
Anti-Topoisomerase I	Scl70
Anticentromere Antibody	ACA
Anti-Mitochondrial Antibody	AMA
Antinuclear Antibody	ANA
Antiphospholipid Syndrome	APS
Antiphospholipid Antibody	APLA
Antisynthetase Antibody	ATSA
Antisynthetase Syndrome	ASS
Aspartate Aminotransferase	AST
Autoimmune Disorders	ADs
Avascular Areas	AAs
CENtromere Protein B	CENP-B
Clinically Suspect Arthralgia	CSA
Connective Tissue Diseases	CTDs
C-Reactive Protein	CRP
Creatinine Phosphokinase	CPK
Dermatomyositis	DM
Diffusion Lung Capacity for Carbon Monoxide	DLCO
Digital Pitting Scars	DPSs
Digital Ulcer	DU
Disease-Modifying Anti-Rheumatic Drugs	DMARDs
Double-Stranded DNA	DsDNA
Eosinophilic Granulomatosis with Polyangiitis	EGPA
Erythrocyte Sedimentation Rate	ESR
Extractable Nuclear Antigen	ENA
Forced Vital Capacity	FVC
Granulomatosis with Polyangiitis	GPA
High-Resolution Computed Tomography	HRCT
Idiopathic Inflammatory Myopathies	IIMs
Idiopathic Pulmonary Fibrosis	IPF
Interstitial Lung Disease	ILD
Interstitial Pneumonia with Autoimmune Features	IPAF
Lactic Dehydrogenase	LDH
Lupus Anticoagulant	LAC
Mechanic’s Hands	MH
Melanoma Differentiation-Associated 5 gene	MDA5
Microscopic Polyangiitis	MPA
Mixed Connective Tissue Disease	MCTD
Multidisciplinary Team	MDT
Myeloperoxidase	MPO
Myositis-Associated Antibodies	MAAs
Myositis-Specific Antibodies	MSAs
Nailfold Videocapillaroscopy	NVC
Nonspecific Interstitial Pneumonia	NSIP
Number of microhEMOrrages	NEMO
Polymyalgia Rheumatica	PMR
Polymyositis	PM
Proteinase-3	PR3
Puffy Hands	PH
Pulmonary Function Tests	PET
Raynaud’s Phenomenon	RP
Rheumatoid Arthritis	RA
Rheumatoid Factor	RF
Ribonucleoprotein	RNP
Schirmer’s Test	ST
Serum Protein Electrophoresis	SPEP
Sjögren’s Syndrome	SjS
Systemic Lupus Erythematosus	SLE
Systemic Sclerosis	SSc
Unstimulated Salivary Flow Rate Test	USFRT
Usual Interstitial Pneumonia	UIP
Very Early Diagnosis of SSc	VEDOSS
White Blood Cell	WBC
